# Joint effect of human papillomavirus exposure, smoking and alcohol on risk of oral squamous cell carcinoma

**DOI:** 10.1186/s12885-023-10948-6

**Published:** 2023-05-19

**Authors:** Zheng Yang, Peng Sun, Kristina R. Dahlstrom, Neil Gross, Guojun Li

**Affiliations:** 1 Department of Otolaryngology Head and Neck Surgery, Key Laboratory of Otolaryngology Head and Neck Surgery of the Ministry of Education, Beijing Tongren Hospital, Capital Medical University, Beijing, 100730 China; 2 Department of Head and Neck Surgery, Unit 1445, The University of Texas MD Anderson Cancer Center, 1515 Holcombe Boulevard, Houston, TX 77030 USA; 3 Department of Otolaryngology, The First Affiliated Hospital of Soochow University, Suzhou, 215006 China; 4 Section of Epidemiology and Population Sciences, Baylor College of Medicine, Houston, TX USA; 5 Department of Epidemiology, The University of Texas MD Anderson Cancer Center, Houston, TX USA

**Keywords:** HPV, Smoking, Alcohol, Cancer risk, Interaction, Oropharyngeal cancer, SCCOC

## Abstract

**Background:**

Smoking, alcohol consumption, and human papillomavirus (HPV) infection are known risk factors for oral squamous cell carcinoma (OSCC) including SCC of oropharynx (SCCOP) and SCC of oral cavity (SCCOC). Researchers have examined each of these risk factors independently, but few have observed the potential risk of their interaction. This study investigated the interactions among these risk factors and risk of OSCC.

**Methods:**

Totally 377 patients with newly diagnosed SCCOP and SCCOC and 433 frequency-matched cancer-free controls by age and sex were included. Multivariable logistic regression was performed to calculate ORs and 95% CIs.

**Results:**

We found that overall OSCC risk was independently associated with smoking (adjusted OR(aOR), 1.4; 95%CI, 1.0–2.0), alcohol consumption (aOR, 1.6; 95%CI, 1.1–2.2), and HPV16 seropositivity (aOR, 3.3; 95%CI, 2.2–4.9), respectively. Additionally, we found that HPV16 seropositivity increased the risk of overall OSCC in ever-smokers (aOR, 6.8; 95%CI, 3.4–13.4) and ever-drinkers (aOR, 4.8; 95%CI, 2.9–8.0), while HPV16-seronegative ever-smokers and ever-drinkers had less than a twofold increase in risk of overall OSCC (aORs, 1.2; 95%CI, 0.8–1.7 and 1.8; 95%CI, 1.2–2.7, respectively). Furthermore, the increased risk was particularly high for SCCOP in HPV16-seropositive ever-smokers (aOR, 13.0; 95%CI, 6.0–27.7) and in HPV16-seropositive ever-drinkers (aOR, 10.8; 95%CI, 5.8–20.1), while the similar increased risk was not found in SCCOC.

**Conclusion:**

These results suggest a strong combined effect of HPV16 exposure, smoking, and alcohol on overall OSCC, which may indicate a strong interaction between HPV16 infection and smoking and alcohol consumption, particularly for SCCOP.

## Introduction

Oral squamous cell carcinoma (OSCC) includes SCC of the oropharynx (SCCOP) and SCC of oral cavity (SCCOC) and affects more than 53,000 men and women each year in the United States, accounting for an estimated 10,860 deaths in the year 2019 [1]. Studies have consistently demonstrated that smoking and alcohol consumption increase the risk of SCC of the head and neck [2]. More recently, epidemiologic and molecular data suggested that infection with human papillomavirus (HPV) is also associated with increased risk of OSCC [3]. Other factors, including genetic factors, oral hygiene, diet, and high-risk sexual behavior, also contribute to the risk of OSCC, suggesting a multifactorial etiology for oral carcinogenesis [2, 4, 5].

Smoking and alcohol drinking are two well-known independent risk factors for OSCC, while smoking may contribute to approximately 25% of all head and neck cancer patients, who are never-drinkers [6, 7]. Although the association of alcohol drinking with OSCC is not as significant as that of smoking, the risk associated with alcohol drinking is in a dose–response relationship and site-specific [5, 7, 8]. Previous studies have shown that smoking and alcohol drinking may have a joint effect on OSCC [5, 6]. Smoking or drinking cessation markedly reduces the risk of OSCC, further emphasizing the significance of these risk factors [5]. Additionally, investigators showed that cigarette smoking increases the incidence of p53 mutations, leading to inactivation of the p53 gene and increased oral tumorigenesis [9]. Alcohol is proposed to play a role in tumorigenesis directly as a carcinogen in its metabolite form (acetaldehyde) or indirectly by causing mucosal damage and leading to increased absorption of other toxins, including those produced in cigarette smoke [9].

In addition to smoking and alcohol use, HPV infection is an independent risk factor for SCCOP [10]. Researchers have found DNA from high-risk HPV types in nearly 30% of OSCC, and high-risk HPV type 16 (HPV16) in particular accounts for more than 90% of HPV-related SCCOP [11]. The incidence of HPV-related SCCOP is increasing, particularly in the young, never-smokers, and never-drinkers, suggesting that the oncogenic nature of HPV contributes to increased incidence of SCCOP despite trends of decreasing tobacco and alcohol use [12–14]. Genomic DNA from high-risk HPV types integrates into host DNA and encode the viral E6 and E7 oncoproteins that degrade and inhibit tumor suppressors p53 and Rb, respectively, providing a clear molecular mechanism of development of HPV-associated malignancy [3]. Because more than 30% of HPV-positive OSCC patients are smokers and drinkers, smoking, alcohol consumption, and HPV infection may have a combined or interactive effect on the risk of OSCC, particularly SCCOP.

Despite recent studies of the incidence of HPV-related OSCC in never-smokers and never-drinkers, the association of HPV infection, smoking, and alcohol consumption with increased risk of OSCC remains controversial. In previous case–control studies of this association, some reported an increased risk of OSCC in the presence of smoking and HPV infection and described a multiplicative effect of heavy alcohol consumption and HPV infection on risk of OSCC [15, 16]. Conversely, others did not observe an increased risk of HPV-positive OSCC with exposure to tobacco or alcohol and suggested two distinct carcinogenic pathways between HPV-related and -unrelated OSCC [10, 17, 18]. In the study described herein, we further confirmed whether HPV exposure combined with smoking and alcohol consumption to increase risk of overall OSCC and stratified analysis by tumor subsites including SCCOP and SCCOC.

## Material and methods

### Study subjects

Patients at The University of Texas MD Anderson Cancer Center were recruited from April 1996 to April 2002 as a part of a molecular epidemiologic study of head and neck squamous cell carcinoma. Eligible patients included those with newly diagnosed, histopathologically confirmed, untreated OSCC. In this study, a total of 377 OSCC patients with different subsites included 211 SCCOP (e.g., base of tongue and tonsils) and 166 SCCOC (e.g., tongue, floor of mouth, palate, buccal mucosa, and gingivae) cases. The accrual rate was 81%. The control participants comprised two groups of cancer-free patients. One group consisted of healthy patients seen for benign conditions at the Kelsey-Seybold Clinic, a multispecialty physician practice with multiple locations throughout the metropolitan Houston area (75% overall response rate). The second group consisted of healthy, genetically unrelated visitors of cancer patients at the outpatient clinics at MD Anderson (80% overall response rate). Eligible controls were individuals with no previous history of cancer except for nonmelanoma skin cancer. Four hundred thirty-three eligible controls were frequency-matched with 377 eligible cases according to age and sex.

Eligible participants completed a questionnaire on demographic characteristics, medical history, and lifetime tobacco and alcohol use. Participants who had smoked more than 100 cigarettes throughout their lifetimes were categorized as ever-smokers, whereas the remaining participants were categorized as never-smokers. Also, participants who had consumed alcoholic beverages at least once a week for more than 1 year were categorized as ever-drinkers, and the remaining were categorized as never-drinkers. The detailed recruitment methods for this case–control study were carried out as described previously [19, 20]. After signing informed consent forms, which had been approved by the institutional review boards (IRB) of both M.D. Anderson Cancer Center and Kelsey-Seybold, study participants completed a questionnaire regarding demographic and relevant risk factors and donated 30 ml of blood for biological testing. All methods were conducted in accordance with the Declaration of Helsinki (1964 and later versions). Written information consent was obtained from all study subjects.

### HPV16 serologic testing

HPV16 L1 virus-like particles generated from recombinant baculovirus-infected insect cells were used to test for antibodies against HPV16 in the plasma of study subjects using a standard enzyme-linked immunosorbent assay [21, 22]. About 10% of the plasma specimens were randomly selected for retesting to confirm the original findings. To eliminate potential binding interference by heparin, the plasma specimens were treated with 43 U/ml heparinase I (Sigma, St. Louis, MO) before testing [23].

#### Tumor HPV16 detection

We extracted DNA from patients’ paraffin-embedded tissue specimens or biopsies and analyzed for the presence of HPV16 using specific polymerase chain reaction (PCR) and in situ hybridization (ISH) assays as described previously [20]. For some patients, the tumor HPV16 status was available from the patients’ clinical records on ISH and p16 immunohistochemical determination, which had become standard practice at the UT MD Anderson's pathology laboratory. Quality control was maintained by re-assaying a subset of samples, and the retested samples matched the original results 100%. 

### Statistical analysis

The differences in the distribution of selected demographic variables (age, sex, ethnicity), smoking, alcohol consumption, and HPV16 status between the cases and controls were evaluated using the χ^2^ test. Both univariate and multivariable logistic regression analyses were used to calculate odds ratios (ORs) and 95% confidence intervals (CIs) for the cases and controls. The stratified analysis was also performed according to tumor site, smoking, drinking, and HPV16 status. In the multivariable logistic regression model, the ORs and 95% CIs were adjusted for age sex, and ethnicity. The combined effect of smoking, alcohol consumption, and HPV16 infection on risk of overall OSCC and stratified tumor subsites evaluated. The similar analysis was further stratified by tumor subsites including SCCOP and SCCOC. All tests were two-sided, with significance defined at *P* levels less than 0.05. All statistical analyses were performed using the SAS software program (version 9.4; SAS Institute, Cary, NC).

## Results

All 377 case subjects with newly diagnosed OSCC and 433 cancer-free control subjects were self-reported individuals. Their demographic variables and risk factors are listed in Table 1. Our analyses illustrated case–control frequency matching according to age and sex (*P* = 0.170 and *P* = 0.393, respectively). Smoking, alcohol consumption, and HPV16 seropositivity were significantly more common in the cases than in the controls (28.9% *versus* 11.4%) (*P* = 0.033, *P* = 0.001, and *P* < 0.001, respectively), particularly in SCCOP (42.6% *versus* 11.4% between SCCOP and SCCOC).

**Table 1:** Demographic variables and risk factors in the OSCC patients and the controls

Variable	Cases (*n* = 377)						Controls (*n* = 433)		P*
	OSCC (All cases)		SCCOP		SCCOC				
Age (years)	n	%	n	%	n	%	n	%	
< 54	158	41.9	97	46.0	61	36.7	161	37.2	.170
≥ 54	219	58.1	114	54.0	105	63.3	272	62.8	
Sex									
Male	280	74.3	173	82.0	107	64.5	310	71.6	.393
Female	97	25.7	38	18.0	59	35.5	123	28.4	
Ethnicity									
Non-Hispanic White	327	86.7	188	89.1	139	83.7	421	97.2	< .001
Others	50	13.3	23	10.9	27	16.3	12	2.80	
Smoking									
Never	115	30.5	72	34.1	43	25.9	146	33.7	.033
Ever	262	69.5	139	65.9	123	74.1	287	66.3	
Alcohol consumption									
Never	88	23.3	42	19.9	46	27.7	152	35.1	.0003
Ever	289	76.7	169	80.1	120	72.3	281	64.9	
HPV16 serology									
Seronegative	268	71.1	121	57.4	147	88.6	379	87.5	< .001
Seropositive	109	28.9	90	42.6	19	11.4	54	12.5	

^*^Two-sided χ^2^ test

The associations of smoking, alcohol consumption, and HPV16 infection with risk of OSCC, SCCOP, and SCCOC are summarized, respectively in Table 2. After adjusting for age, sex, ethnicity, alcohol and HPv16 serology, smoking was associated with increased risk of OSCC independent of alcohol use and HPV16 status (aOR, 1.4; 95% CI, 1.0–2.0). Similarly, alcohol consumption was independently associated with increased risk of OSCC (aOR, 1.6; 95% CI, 1.1–2.2). HPV16 seropositivity was more significantly associated with increased risk of OSCC (aOR, 3.3; 95% CI, 2.2–4.9) than were smoking and alcohol use, particularly for SCCOP (aOR, 6.6; 95% CI, 4.2–10.3) compared with SCCOC (aOR, 1.0; 95% CI, 0.5–1.6).

**Table 2:** Effect of smoking, alcohol consumption, and HPV16 serology on risk of OSCC, stratified by tumor sites

Risk factors	Controls (*n* = 433)		Cases (*n* = 377)								
	n	%	OSCC (All cases)			SCCOP			SCCOC		
			n	%	aOR^a^ (95% CI)	n	%	aOR^a^ (95% CI)	n	%	aOR^a^ (95% CI)
	433	100	377	100		211	56.0		166	44.0	
Smoking											
Never	146	33.7	115	30.5	1.0	72	34.1	1.0	43	25.9	1.0
Ever	287	66.3	262	69.5	1.4 (1.0–2.0)	139	65.9	1.6 (1.1–2.6)	123	74.1	1.4 (1.1–2.2)
Alcohol consumption											
Never	152	35.1	88	23.3	1.0	42	19.9	1.0	46	27.7	1.0
Ever	281	64.9	289	76.7	1.6 (1.1–2.2)	169	80.1	1.7 (1.1–2.7)	120	72.3	1.4 (1.1–2.3)
HPV16 status											
Seronegative	379	87.5	268	71.1	1.0	121	57.4	1.0	147	88.5	1.0
Seropositive	54	12.5	109	28.9	3.3 (2.2–4.9)	90	42.6	6.6 (4.2–10.3)	19	11.5	1.0 (0.5–1.6)

^a^aORs: Adjusted ORs by age, sex, ethnicity, and/or smoking, alcohol consumption, and HPV16 serology in logistic regression models

Because smoking, alcohol consumption, and HPV16 infection appeared to be independently associated with increased risk of OSCC, and because there was a clear difference between SCCOC and SCCOP with respect to HPV-16 L1 serostatus and cancer risk (Table 2), any further analysis was performed separately for these groups. Thus, we performed a multivariable analysis of the interaction of these three variables and its effect on risk of OSCC, and such an analysis was further stratified by tumor subsites. As shown in Table 3, after adjusting for age, sex, and ethnicity, we observed significantly greater OSCC risk in patients who were HPV16-seropositive and ever-smokers (aOR, 6.8; 95% CI, 3.4–13.4) or ever-drinkers (aOR, 4.8; 95% CI, 2.9–8.0) than in those who were HPV16-seropositive and never-smokers (aOR, 2.1; 1.2–3.6) or never-drinkers (aOR: 2.3; 1.7–8.8). In comparison, the risk of OSCC in patients who were HPV16-seronegative and ever-smokers or ever-drinkers was less than twofold (aOR, 1.2; 95% CI: 0.8–1.7 and 1.8, 95% CI, 1.2–2.7, respectively). Furthermore, such significantly increased risk was even higher in SCCOP patients who were HPV16-seropositive and ever-smokers (aOR, 13.0; 95% CI, 6.0–27.7) or ever-drinkers (aOR, 10.8; 95% CI, 5.8–20.1) than in those who were HPV16-seropositive and never-smokers (aOR, 4.9; 2.6–9.2) or never-drinkers (aOR: 5.1; 3.0–20.7) (Table 3). In contrast, risk for SCCOP in patients who were seronegative was not increased among ever smokers and only slightly so among ever drinkers (aOR, 1.4; 95% CI: 0.8–2.4 and 1.8, 95% CI, 1.2–4.1, respectively; Table 3). Among SCCOC patients, we did not observe significant interactions between L1 serostatus and smoking or alcohol, except for a slightly protective effect of seropositivity among never-smokers and increased risk among HPV16-seropositive ever-smokers (Table 3).

**Table 3:** Combined effect of smoking, alcohol consumption, and HPV16 serology on risk of OSCC, stratified by tumor sites

Risk factors	HPV16 status	Controls (*n* = 433)		Cases (*n* = 377)								
		n	%	OSCC (All cases)			SCCOP			SCCOC		
				n	%	aOR^a^ (95% CI)	n	%	aOR^a^ (95% CI)	n	%	aOR^a^ (95% CI)
							211	56.0		166	44.0	
Smoking												
Never	−	106	24.5	65	17.2	1.0	27	12.8	1.0	38	22.9	1.0
	+	40	9.2	50	13.3	2.1 (1.2–3.6)	45	21.3	4.9 (2.6–9.2)	5	3.0	0.3 (0.1–0.9)
Ever	−	273	63.1	203	53.9	1.2 (0.8–1.7)	94	44.6	1.4 (0.8–2.4)	109	65.7	1.2 (0.7–1.9)
	+	14	3.2	59	15.6	6.8 (3.4–13.4)	45	21.3	13.0 (6.0–27.7)	14	8.4	2.5 (1.0–6.1)
Alcohol consumption												
Never	−	137	31.6	60	15.9	1.00	22	10.4	1.0	38	22.9	1.0
	+	15	3.5	28	7.4	2.3 (1.7–8.8)	20	9.5	5.1 (3.0–20.7)	8	4.8	1.5 (0.6–4.0)
Ever	−	242	55.9	208	55.2	1.8 (1.2–2.7)	99	46.9	1.8 (1.2–4.1)	109	65.7	1.4 (0.8–2.2)
	+	39	9.0	81	21.5	4.8 (2.9–8.0)	70	33.3	10.8 (5.8–20.1)	11	6.6	0.8 (0.3–1.8)

^a^aORs: Adjusted by age, sex, ethnicity and/or smoking, alcohol consumption, and HPV16 serology in logistic regression models

## Discussion

In this case–control analysis, more patients with OSCC than cancer-free controls had a history of smoking, alcohol consumption, or HPV infection. We found that smoking, alcohol consumption, and HPV16 infection independently increased the risk of OSCC, particularly for SCCOP. Smoking and alcohol use as the traditional OSCC risk factors were independently associated with a moderately increase in risk of OSCC, and HPV16 seropositivity was associated with a great increase in risk of it, which were consistent with results of prior studies [10, 24]. However, the results of previous investigations have been unclear regarding the combined effect of HPV exposure with tobacco and alcohol use on risk of OSCC and their tumor subsites. In this combined study of HPV exposure with traditional OSCC risk factors, we found that among patients who were HPV16-seropositive, ever-smokers and -drinkers had a markedly greater risk of OSCC than did never-smokers and -drinkers, particularly among those with SCCOP patients**.**

In a study of HPV DNA in exfoliated oral cells, Smith et al. reported a synergistic effect of heavy alcohol consumption and HPV infection on risk of OSCC but did not observe such an effect of smoking and HPV infection [16]. Alternatively, others observed an increased risk of SCCOP patients who were seropositive for anti-HPV16 antibodies and had tobacco exposure [15, 25, 26]. Other studies of the interactions among tobacco and alcohol use and HPV16 infection demonstrated no evidence of synergistic interactions [10, 24–29]. In the present study, we observed a strong combined effect of HPV16 seropositivity with tobacco or alcohol use on risk of OSCC, particularly for SCCOP, while such combined effect was not found in SCCOC. This suggests that the carcinogenic effects of smoking and alcohol consumption and the genomic instability caused by HPV infection and p53 inactivation act at different tumor sites and stages of carcinogenesis, resulting in a multiplicative rather than additive effect on tumorigenesis.

One possible reason for recent disparate conclusions in the literature regarding the interaction among risk factors for OSCC is use of different methodologies of HPV detection combined with different types of HPV. HPV markers can be detected as HPV DNA in exfoliated oral cells and biopsy specimens and as anti-HPV antibodies in serum. Smith et al. examined HPV DNA in exfoliated oral cells and found that detection of HPV DNA in them was associated with increased risk of OSCC [16], whereas Herrero et al. found no association between HPV DNA in exfoliated cells and detection of HPV DNA in oral tumor biopsy specimens [27]. Other studies support the findings described by Herrero and colleagues, suggesting that HPV DNA in exfoliated cells is a poor marker of tumor HPV status [15]. In addition, Gillison et al. stratified head and neck squamous cell carcinomas according to tumor HPV status and found no associations among tobacco and alcohol use and HPV16-positive tumor biopsy specimens [28]. We conceded that detection of anti-HPV16 antibodies in serum suggests exposure with HPV at any mucosal site, not specifically the oral mucosa, and HPV16 L1 serology is a marker for past infection and may not be associated with the tumor HPV status. Also, whereas anti-HPV antibodies generally indicate invasive HPV infection, some individuals may not undergo seroconversion. Additionally, antibody levels may not be maintained over time, making anti-HPV16 antibodies in serum difficult markers to interpret. Nonetheless, antibodies are minimally invasive markers predictive of exposure to HPV and invasive HPV infection [30]. In this current study, HPV L1 seropositivity was similar among controls and patients with SCCOC (12.5% *vs.* 11.4%), while almost 43% of SCCOP patients were seropositive. Given the difference between SCCOC and SCCOP, we analyzed these separately. Moreover, our additional analysis found a significant association and correlation between HPV16 L1 serology and tumor HPV16 status among 156 SCCOP patients whose tumor HPV16 status are available (aOR, 3.4 and 95% CI, 1.5–7.8 and correlation efficient: *r* = 0.262 and *p* = 0.001) (Table 4). Nevertheless, we will validate these findings in our future larger study.

**Table 4:** Association and correlation between HPV16 L1 serology and HPV16 status in tumor tissue among 156 SCCOP patients

HPV16 serology	Tumor HPV16 status				
	HPV16 (-) cases (*n* = 89)		HPV16( +) cases (*n* = 67)		Adjusted OR^a^, 95% CI
	N	%	N	%	
L1 serology (-)	76	85.4	42	62.7	1.0
L1 serology ( +)	13	14.6	25	37.3	3.4
Correlation	Correlation efficient; *r* = 0.262 and *p* = 0.001				

^a^ORs were adjusted for age, sex, ethnicity, tobacco smoking and alcohol drinking in logistic regression model

Discrepancies in the findings describing the interactions among HPV infection, smoking, and alcohol consumption may also be related to different subsites of OSCC, such as the oral cavity (tongue, floor of mouth, palate, buccal mucosa, and gingivae) and oropharynx (base of tongue and tonsils), which are associated with different cancer risk factors. In a pooled case–control series, Lubin et al. demonstrated similarly increased risk of SCCOC and SCCOP with smoking and drinking [8]. This is consistent with historical evidence that smoking and alcohol consumption are strongly associated with OSCC at all subsites. Conversely, the rate of infection with HPV has varied depending on the head and neck cancer subsite. Consistent with previous meta-analyses [31, 32]. For example, in a study of 92 head and neck squamous cell carcinomas patients, their findings demonstrated that patients with SCCOP had a higher HPV infection rate than did patients with SCCOC [33]. These results suggest that stratification of patients according to head and neck cancer subsite is important in classifying specific cancer risk and associated risk factors.

In this case–control study, we controlled for the confounding variables of, age and sex by frequency-matched on these variables and adjusted with these variables with additional ethnicity to HPV exposure between the controls and cases, who had not undergone treatment. The limitations of this hospital-based study include possible selection bias due to the selection of cases and controls from different populations. Another limitation of this study is the use of L1 serology as a marker for an HPV-related cancer. The presence of L1 antibodies indicates past exposure to HPV16 that may not necessarily be related to an oral HPV infection or HPV-related tumor, while we indeed observed high L1 seropositivity among SCCOP patients relative to both cancer-free controls and SCCOC patients (not typically HPV-related). Therefore, we believe that L1 may serve as a potential surrogate marker for an HPV-related SCCOP in this population. Also, small sample sizes may also limit the statistical power of data analysis, which could restrict the generalizability of the findings. Furthermore, although the use of HPV serology enabled us to use a cancer-free control group, HPV serology we used is not specific to OSCC tumor status but is rather a reasonable indication of HPV exposure [27, 30]. Infection with high-risk HPV types other than HPV16 may also be associated with OSCC, but we limited this study to anti-HPV16 antibodies. Finally, patients’ self-reporting of previous alcohol and tobacco use may have resulted in recall bias, in which case the estimates of association between smoking and alcohol consumption exposure alone or in combination with HPV exposure and risk of OSCC may have been underestimated.

## Conclusion

Whether smoking and alcohol use are associated with higher risk in HPV positive OSCC, particularly for SCCOP remains unclear. This is one of few case‐control studies to investigate the role of tobacco smoking, alcohol drinking and oral HPV infection in development of OSCC with further stratification of tumor subsite. The combined tobacco and alcohol with HPV16 exposure produced a significant synergistic effect on the risk of OSCC, particularly for SCCOP rather than SCCOC. Oral infection with HPV16 exposure increased the risk of OSCC in ever smokers and ever drinkers, and the effects of tobacco and alcohol were substantially higher in HPV16 seropositive than in HPV16 seronegative OSCC, particularly for SCCOP. This multiplicative effect of combined risk factors suggests that HPV infection and tobacco and alcohol carcinogens may affect different steps in multistep tumorigenesis for OSCC, especially for SCCOP. Further investigations are warranted notably on the interaction of these three risk factors in well-designed, larger, and prospective studies.

## Data Availability

The data underlying this article will be shared on reasonable request to the corresponding author.

## Abbreviations

CI: Confidence interval
HPV: Human papillomavirus
HPV16: Human papillomavirus type 16
OSCC: Oral squamous cell carcinoma
SCCOP: SCC of oropharynx
SCCOC: SCC of oral cavity
OR: Odds ratio
